# Object memory is multisensory: Task-irrelevant sounds improve recollection

**DOI:** 10.3758/s13423-022-02182-1

**Published:** 2022-09-27

**Authors:** Shea E. Duarte, Simona Ghetti, Joy J. Geng

**Affiliations:** 1 Center for Mind and Brain, University of California, Davis, 267 Cousteau Pl, Davis, CA 95616 USA; 2 Department of Psychology, University of California, Davis, Davis, CA USA

**Keywords:** Recognition memory, Dual-process models, Multisensory, Audiovisual

## Abstract

Hearing a task-irrelevant sound during object encoding can improve visual recognition memory when the sound is object-congruent (e.g., a dog and a bark). However, previous studies have only used binary old/new memory tests, which do not distinguish between recognition based on the recollection of details about the studied event or stimulus familiarity. In the present research, we hypothesized that hearing a task-irrelevant but semantically congruent natural sound at encoding would facilitate the formation of richer memory representations, resulting in increased recollection of details of the encoded event. Experiment 1 replicates previous studies showing that participants were more confident about their memory for items that were initially encoded with a congruent sound compared to an incongruent sound. Experiment 2 suggests that congruent object-sound pairings specifically facilitate recollection and not familiarity-based recognition memory, and Experiment 3 demonstrates that this effect was coupled with more accurate memory for audiovisual congruency of the item and sound from encoding rather than another aspect of the episode. These results suggest that even when congruent sounds are task-irrelevant, they promote formation of multisensory memories and subsequent recollection-based retention. Given the ubiquity of encounters with multisensory objects in our everyday lives, considering their impact on episodic memory is integral to building models of memory that apply to naturalistic settings.

**Supplementary Information:**

The online version contains supplementary material available at 10.3758/s13423-022-02182-1.

## Introduction

Multisensory events are ubiquitous in natural environments, and the integration of crossmodal signals has effects that cascade from perception to learning (Shams & Seitz, 2008; Stein et al., 2020). Despite the prevalence and influence of multisensory stimuli, most areas of research in cognition adopt a unisensory perspective, including memory. For example, studies of recognition memory have traditionally used lists of words or objects presented in a single modality. However, recent research has shown that audiovisual presentations of objects along with their characteristic sounds can improve later object recognition memory (Heikkilä, Alho, Hyvönen, & Tiippana, 2015; Lehmann & Murray, 2005; Matusz et al., 2017; Moran et al., 2013; Thelen et al., 2015). This underscores the importance of understanding how multisensory perceptual events impact the formation of specific memories.

In an early demonstration of the multisensory advantage in memory, Lehmann and Murray (2005) had participants discriminate between old and new objects that were initially visual-only or presented with an object-congruent or object-incongruent simultaneous sound. Accuracy on “old” trials was higher for objects initially paired with a congruent sound, despite the sounds being completely task-irrelevant. In subsequent studies, multisensory “old” trials were also differentiated via greater BOLD activation in the lateral occipital cortex (Murray et al., 2005), and event-related potential (ERP) results showed distinct brain networks involved as early as 60–135 ms post-visual stimulus (Murray et al., 2004). The emerging work in this area highlights the impact of multisensory processing on recognition memory; however, the binary old/new recognition tasks employed in these studies have led to findings that lack specificity as to which memory mechanisms are affected by multisensory presentations. In the present research, we consider how multisensory processing affects two forms of object memory: recollection and familiarity.

Dual-process memory models posit that recognition memory depends on the contribution of two behaviorally and neurally distinguishable processes, namely recollection and familiarity (Diana et al., 2007; Yonelinas, 2002; Yonelinas et al., 2010; but see Wais et al., 2006). Recollection reflects the retrieval of specific information from an episodic event, such as when or where the event occurred (e.g., recalling from where you know someone you see on the street), whereas familiarity reflects a general measure of memory strength (e.g., knowing you have seen the person before) (Yonelinas, 2002; Yonelinas et al., 2010). Research suggests that encoding manipulations can differentially affect recollection or familiarity. One such situation is the *congruency effect*, whereby an encoded noun is better remembered when paired with a semantically congruent adjective (e.g., banana-yellow) than an incongruent adjective (e.g., spinach-ecstatic) ( Atienza et al., 2011; Craik & Tulving, 1975; Hashtroudi, 1983). Bein et al. (2015) showed a higher proportion of subjective “recollection” responses to items encoded in the semantically congruent condition, coupled with enhanced retrieval of the context word itself, whereas “familiarity” responses did not differ between conditions. Although the Bein et al. (2015) study used pairs of visually encoded words, studies of multisensory effects on object memory using semantically congruent images and natural sounds may similarly yield recollection-specific memory benefits. On the other hand, increasing the perceptual fluency of stimuli during encoding can support both recollection and familiarity (Yonelinas, 2002). Chen and Spence (2010) showed that multisensory processing of congruent image-sound pairs facilitated the identification of visual objects that were perceptually degraded by visual masks. If the memory benefit of multisensory processing is due solely to improved identification and perception of the visual object during study, both recollection and familiarity may be expected to improve; familiarity may even benefit more than recollection given its relation to priming mechanisms supporting object identification (e.g., Wang & Yonelinas, 2012).

In the present research, we aim to replicate experimental findings demonstrating the benefits of task-irrelevant, congruent sounds on object memory, and delineate whether this effect is driven by improvements to recollection or familiarity-based recognition. We use experimental paradigms derived from the memory literature to address methodological limitations of previous work on multisensory memory. These prior studies have used binary old/new memory tests, from which a single hit rate and false alarm rate are obtained for items in each condition. However, collecting multiple hit and false alarm rates per participant and encoding condition is essential to measure latent memory signals accurately because hit rates alone are susceptible to response biases that obscure the true strength of the underlying memory trace (Brady et al., 2021; Macmillan & Creelman, 1990). For example, a participant might adopt a very stringent criterion and only endorse an item as old if they are very confident and can retrieve many details. We address this limitation by collecting confidence ratings with each old/new recognition response to examine hit and false alarm rates across a range of response criteria (i.e., confidence levels) for each participant and encoding condition.

Our central hypothesis was that hearing a task-irrelevant but semantically congruent natural sound at encoding would facilitate the formation of a richer memory representation that would support recollection of details of the encoded event. To anticipate our results, in Experiment 1, we replicated findings showing improved recognition memory for visual images of objects originally presented with a congruent sound compared to those presented with an incongruent sound. In Experiment 2, we formally measured both recollection and familiarity-based recognition, and found that congruent sounds during encoding specifically supported recollection-based recognition. Finally, in Experiment 3, we asked participants to recollect the sound that was associated with each image at encoding (congruent, control, incongruent) and found the highest rates of recollection for objects seen in a congruent audiovisual pair at encoding. Across three experiments using different methods to estimate recollection and familiarity, we found converging evidence that congruent multisensory information during encoding enhances subsequent recollection.

## Experiment 1

We first aimed to conceptually replicate previous studies showing generally improved recognition memory for congruent multisensory pairs using a blocked design and a surprise memory task including confidence ratings. Participants completed a within-subjects audiovisual encoding task in which visual items were paired with congruent, incongruent, or meaningless control sounds, followed by a visual-only, surprise recognition test. Importantly, the auditory stimuli had no relevance for the encoding task, which was to determine if the visual object would fit into a standard-size suitcase, and participants were not asked to remember the items. Half of the visual images were overlaid with semi-transparent visual noise during the encoding task that degraded the visibility of the object. This manipulation served to avoid memory ceiling effects (see Heikkilä et al., 2015) and to test whether multisensory processing supports memory by improving the perceptual fluency of visually obscured items at encoding. The memory task included four response options to assess whether semantically congruent audiovisual pairs led to higher-confidence recognition memory than incongruent or control pairs, and to calculate hit and false alarm rates for each confidence level to examine the effect of congruency across response criteria.

### Method

#### Participants

Seventy-five students (62 identified as female and 13 identified as male, *M*_*age*_ = 19.8 years) from the University of California, Davis, participated in exchange for partial course credit. Nine participants were excluded based on our pre-registered exclusion criteria due to low accuracy (below chance, 50%) on either the encoding task or the recognition memory task. We also administered a debriefing questionnaire, which was used to determine whether participants should be excluded due to a noisy testing environment, exerting little or no effort in completing the study, or a lack of access to consistent audio (due to glitches, volume changes, or a lack of working speakers) (see Online Supplemental Materials (OSM) for full list of questions). No participants were excluded in Experiment 1 under these criteria. Our sample size was determined with an a priori power analysis using the python package Pingouin (Vallat, 2018) with power (1-β) set at 0.95 and α = 0.05. Prior unpublished data from our laboratory showed an effect of initial sound congruency on recognition memory for visual items with an effect size of *η*_*p*_^*2*^ = 0.06, which requires at least 33 participants to detect. To account for poor testing conditions associated with online data collection, we doubled this number to 66, and data were collected until we reached this point post-exclusion. The pre-registered sample size and exclusion criteria can be found on the Open Science Framework (https://osf.io/5uz24/).

#### Materials

A total of 180 images of three-dimensional (3D) models of common objects (i.e., tools, household objects, vehicles, animals, instruments, recreational equipment, and miscellaneous common items; see OSM for a full list of items) were gathered from the Unity Asset Store (https://assetstore.unity.com/3d). Using the Unity Editor, objects were rotated to easy-to-recognize orientations and edited to reflect the position the object typically assumes when making a sound in order to improve the perception of unity between the item and the sound (e.g., the dog model was edited to have an opened mouth, as if it were barking) (Edmiston & Lupyan, 2015). We used the python package scikit-image (Van der Walt et al., 2014) to remove the image backgrounds, convert them to black and white, and size them to the same dimensions (500 × 500 pixels). The real-world sizes of half of the objects in the images were “small” (small enough to easily fit in a standard suitcase) and the other half were “large.” Ninety of these were used in the encoding task, and 90 new objects from the same categories were integrated into the recognition memory task for a total of 180 items. New items in the recognition task were selected from the same categories as the old objects, and because they make similar types of sounds as the old items either on their own (e.g., a rabbit) or when interacted with (e.g., a scooter) (see OSM). The old and new items were not counterbalanced across the encoding and recognition tasks, but importantly, the old items, which all had associated sounds, were counterbalanced across the six encoding conditions across participants. Thus, while overall recognition discrimination between old and new objects may be different, this would not affect the critical comparison of interest between recognition of items paired with different sounds in the “congruent,” “incongruent,” or “white noise” encoding conditions. Six different visual masks were manually created using a variety of black, white, and gray geometric shapes arranged in a square the same size as the images. These were used at 100% opacity for the post-stimulus mask, and displayed at 50% opacity when overlaid on top of images as visual noise.

Natural sounds and white noise sounds were obtained from the Multimost Stimulus Set (Schneider, Engel, & Debener, 2008) or found on https://findsounds.com/. Ninety natural sounds corresponded to the items in the encoding task for the congruent condition, 15 variations of white noise were used for the control condition, and a separate set of 30 natural sounds were used for items in the incongruent condition. The same 30 incongruent sounds were used in every version of the experiment, and were chosen from the same categories as the visual objects. These were all from the Multimost Stimulus Set, from which all sounds were shown to be identifiable on their own (Schneider et al., 2008). For each version of the experiment, incongruent sounds were randomly paired with visual objects, and these pairs were manually rearranged in cases where the visual object could be expected to make a noise that was at all similar (e.g., the whistle sound would not be paired with the bird image) (see OSM for a full list of images, sounds, and combinations used). All sounds were 400 ms in length and amplitude normalized using Audacity (Audacity Team, 2021).

#### Procedure

Participants completed separate encoding and recognition blocks online via personal computers through the online stimulus presentation software Testable (https://www.testable.org/). Before the encoding task began, a string of sample beeps was played, and participants were asked to adjust their sound level to a comfortable volume and not to alter it for the remainder of the study.

##### Encoding block

**Fig. 1:**
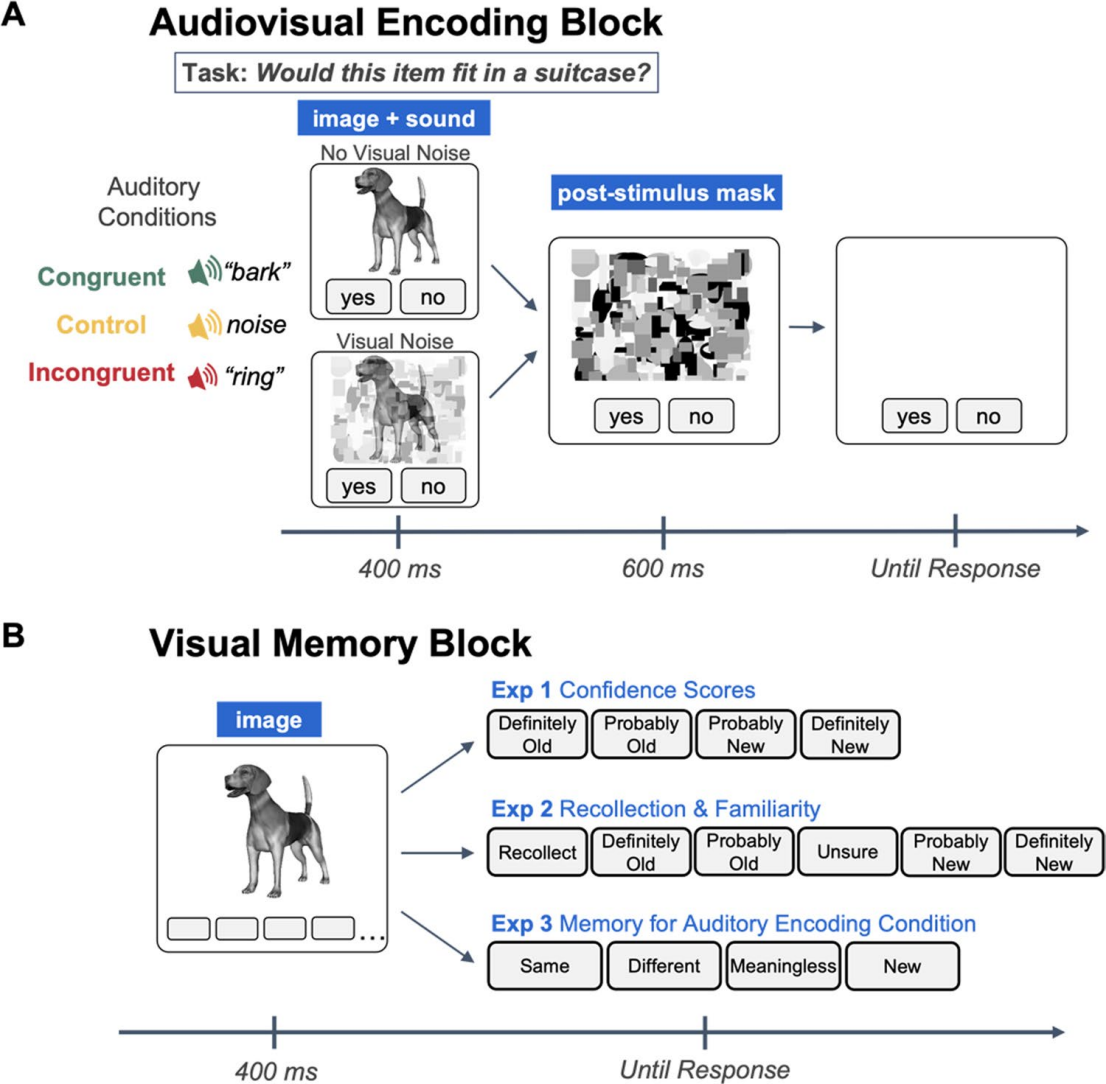
**a** The audiovisual encoding task used for all three experiments. Experiments 1 and 3 include the visual noise manipulation during the initial 400-ms presentation, while in Experiment 2, all presentations are overlaid by the geometric visual noise during this period. **b** Surprise visual memory tasks for all three experimentsa

The encoding block consisted of a size judgment task. Ninety object-sound pairs were presented during this block (30 congruent pairs, 30 control pairs, and 30 incongruent pairs). On each trial, a visual and an auditory stimulus were simultaneously presented for 400 ms, followed by a 600-ms post-stimulus mask. The post-stimulus mask functioned to limit continued visual processing of the object in order to accentuate the timing co-occurrence of the visual and auditory stimuli (Kinsbourne & Warrington, 1962). Participants were to respond by clicking an on-screen “yes” button if the visually presented item would fit inside a standard-sized suitcase, and “no” if it would not. Importantly, participants were informed not to pay attention to the sounds, and to base their size judgments on the item in the image. The auditory stimulus was semantically congruent with the visual stimulus, incongruent, or a white noise control sound. Additionally, while all presentations were followed by the 600-ms post-stimulus visual mask, half of the items were also overlaid with visual noise during the initial 400-ms presentation. Therefore, there were three levels of Auditory Condition (congruent, incongruent, and control), and two levels of Visual Noise (visual noise, no visual noise) (see Fig. 1a). Visual stimuli were counterbalanced across the six, within-subjects encoding conditions, and there were 90 trials randomized for each participant. The size judgment task was designed to prevent participants from expecting that their memory might be tested for objects in this block, making the recognition block a test of incidental memory.

##### Recognition block

Immediately following the encoding block, participants completed a visual-only surprise recognition task. In this task, the 90 old images were intermixed with 90 new images for a total of 180 trials. On each trial, a visual stimulus was presented for 400 ms, and participants gave a confidence-based recognition response, indicating whether the item was “definitely old,” “probably old,” “probably new,” or “definitely new” (see Fig. 1b). Trials were randomized for each participant.

##### Debriefing questionnaire

After the experiment, participants responded to questions on a debriefing survey, which allowed us to assess the quality of the testing environment and stimulus presentation. The questionnaire included questions about the testing environment, the subjective volume and quality of the auditory stimuli, whether the volume was adjusted during the experiment, and whether any glitches or lags between audiovisual stimuli were experienced, among others. As this experiment was completed remotely, responses were used to exclude participants when the testing environment or stimulus presentations were not of adequate quality.

#### Data analysis

The design, hypotheses, and statistical analyses for Experiment 1 were preregistered prior to data collection on the Open Science Framework, and the raw data files and analysis code are publicly available (https://osf.io/5uz24/). The preregistered analysis tests for differences in memory performance (indexed by confidence scores) between encoding conditions. We also performed an exploratory receiver operating characteristic (ROC; Yonelinas & Parks, 2007) analysis to assess hit and false alarm rates between encoding conditions at each response criterion. Additionally, we have included mean accuracy (% correct) for the encoding and recognition tasks in Table 1, and recognition accuracy across categories can be found in the OSM.

**Table 1:** Mean accuracy for the encoding and recognition tasks and recognition confidence scores for items in each Auditory and Visual Noise condition from Experiment 1. Chance performance is 50% for both encoding and recognition tasks

	Encoding task accuracy (% correct)		Recognition task accuracy (% correct)		Recognition confidence scores	
Auditory Condition	No visual noise	Visual noise	No visual noise	Visual noise	No visual noise	Visual noise
Congruent	0.84(0.36)	0.79(0.41)	0.86(0.34)	0.72(0.45)	3.62(0.76)	3.18(1.05)
Control	0.84(0.37)	0.79(0.41)	0.86(0.34)	0.70(0.46)	3.58(0.80)	3.13(1.04)
Incongruent	0.81(0.39)	0.75(0.43)	0.85(0.35)	0.66(0.47)	3.56(0.78)	3.00(1.09)

Standard deviations are shown in parentheses

##### Recognition confidence scores

First, consistent with our preregistered approach to compare the strength of recognition confidence on old items between conditions, we transformed each response option to a numerical value representing its relative strength (i.e., “definitely old”: 4, “probably old”: 3, “probably new”: 2, and “definitely new”: 1). For old trials in the recognition block, we performed a 2 (Visual Noise: visual noise vs. no visual noise) × 3 (Auditory Condition: congruent, control, incongruent) repeated-measures analysis of variance (RM ANOVA) on these confidence scores, and post hoc t-tests with Bonferroni-adjusted alpha levels were used for pairwise comparisons. Bayes factors were also computed for pairwise comparisons to consider the weight of evidence for the tested hypotheses, and interpreted in accordance with Lee and Wagenmakers (2013).

##### ROC analysis

The analysis of confidence scores suggests that items belonging to one experimental condition are recognized with higher confidence than those belonging to another group. However, as illustrated in the *Introduction*, an analysis based on hit rates alone is liable to obscure the true nature of latent memory signals. To better characterize the underlying memory signals in each condition, we calculated the hit rates (the proportion of old items correctly identified as old) and false alarm rates (the proportion of new items incorrectly identified as old) for items in each Auditory Condition at each of our four response options to analyze the underlying ROC (Yonelinas & Parks, 2007). Each subsequent point on an ROC curve relates the hit and false alarm rates as participants increasingly relax their criteria for classifying an item as “old,” from “definitely old” to “definitely new.” Therefore, the leftmost point of each ROC reflects the hit and false alarm rates for trials on which participants responded “definitely old,” the second point from the left reflects the hit and false alarm rates for trials on which participants chose either “definitely old” or “probably old,” and so on. The rightmost points have been excluded from the figures because the cumulative hit and false alarm rates converge to one at these points.

**Fig. 2:**
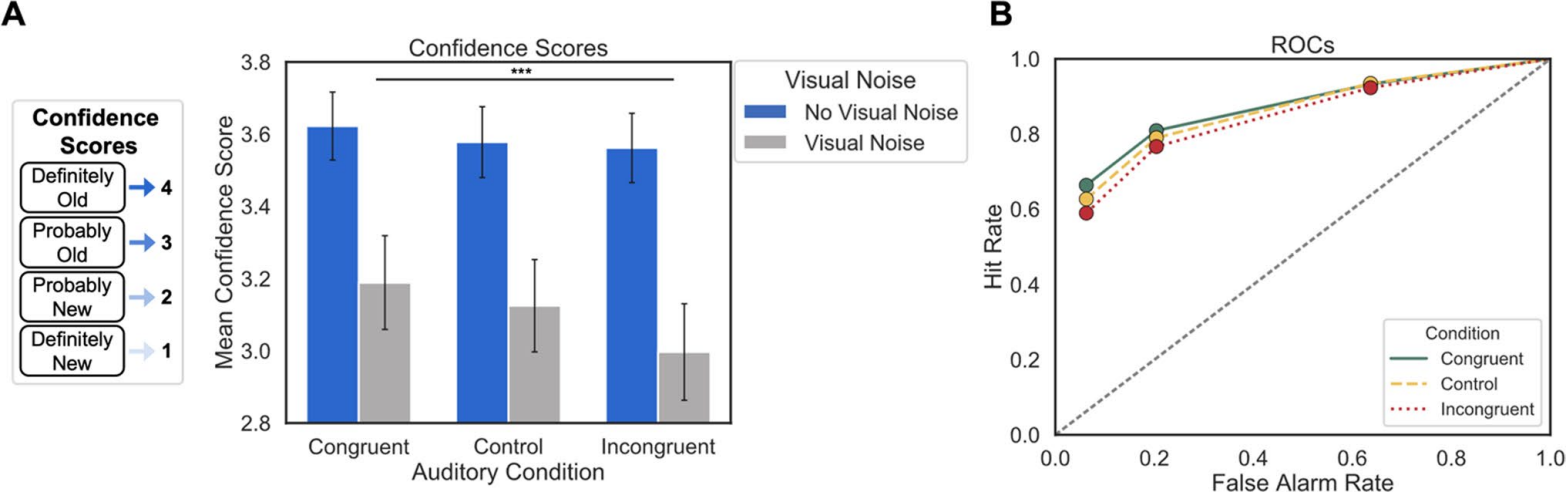
**a** Mean confidence scores for items in each Visual Noise and Auditory Condition from Experiment 1. The box to the left of the graph illustrates the translation of response options to confidence scores. Error bars denote standard error of the mean. Average confidence scores are higher for items encoded in the congruent than incongruent Auditory Condition, and for items encoded with no visual noise than with visual noise. **b** Average observed receiver operating characteristic curves (ROCs) for each Auditory Condition in Experiment 1, collapsed across Visual Noise Conditions. Each successive point (from left to right) on a given ROC represents the cumulative hit and false alarm rate for items in that condition during the recognition task. Memory performance (AUC) is greater for congruent than incongruent items, and the greatest differences in performance occur at the highest confidence level (leftmost point)

For statistical analyses, individual ROCs were constructed for each participant at each level of Auditory Condition, and the points in Fig. 2b reflect the average observed hit and false alarm rates at each response option across participants. Because we did not observe a significant interaction between Visual Noise and Auditory Condition in our primary analysis, we collapsed across levels of Visual Noise to construct ROCs with a greater number of observations per condition. To compare overall recognition memory strength between Auditory Conditions, we calculated the area under the curve (AUC) of each participant’s observed ROCs, which is a theoretically agnostic metric of performance, where a greater area under the curve indicates better recognition memory performance. We performed a repeated-measures ANOVA and Bonferroni-adjusted post hoc pairwise t-tests. We note that there were too few response options to fit these ROC data to the dual-process signal detection model, which we address in Experiment 2.

### Results

#### Recognition confidence scores

A repeated-measures ANOVA showed a significant main effect of Auditory Condition on recognition confidence scores (Fig. 2a), *F*(2, 130) = 8.41, *p <* 0.001, *η*_*p*_^*2*^ = 0.12, such that confidence scores were higher for items encoded in the congruent condition than in the incongruent condition, *t*(65) = -3.92, *p* < 0.001 (see Table 1). The Bayes factor indicated very strong evidence for this finding (*BF*_10_ = 105.51). This is consistent with our main hypothesis that stronger memories are formed for visual objects initially paired with congruent compared to incongruent sounds. Post hoc t-tests did not reveal significant differences between confidence scores for items in congruent and control conditions, or control and incongruent conditions, *t*(65) = -1.81, *p* = 0.23; *t*(65) = 2.36, *p* = 0.06. Nevertheless, Bayes factors did not provide evidence for the null hypotheses for the former comparison (*BF*_01_ = 1.59) and none for the latter (*BF*_01_ = 0.56). There was also a main effect of Visual Noise, *F*(1, 65) = 269.05, *p* < 0.001, *η*_*p*_^*2*^ = 0.81, with higher confidence scores for items with no visual noise than with visual noise. There was no significant interaction between Auditory Condition and Visual Noise, *F*(2, 130) = 2.72, *p* = 0.07.

#### ROC analysis

A one-way repeated-measures ANOVA on AUC for individual ROC curves (Fig. 2b) revealed a significant effect of Auditory Condition, *F*(2, 130) = 5.01, *p =* 0.008, *η*_*p*_^*2*^ = 0.07, post hoc t-tests showed that memory performance was better for congruent items (*M* = 0.82, *SD* = 0.09) than incongruent items (*M* = 0.80, *SD* = 0.10), *t*(65) = 3.02, *p* = 0.01, with the Bayes factor providing moderate evidence for this finding (*BF*_10_ = 8.17) (Fig. 2b). AUC was not significantly different between congruent and control items (*M* = 0.81, *SD* = 0.10) or between control and incongruent items, *t*(65) = 1.32, *p* = 0.57; *t*(65) = 1.88, *p* = 0.19, and Bayes factors provide only moderate evidence for the null hypothesis former comparison (*BF*_01_ = 3.23) and anecdotal for the latter (*BF*_01_ = 1.41). This pattern of data is consistent with the Confidence Score analysis and shows recognition memory was greater for the congruent than the incongruent Auditory Conditions even when response rates are corrected by false alarms at each confidence level. This analysis further illustrates that the difference between conditions decreases as the response criteria relaxes, which indicates that the Auditory Condition primarily affects whether participants recognize items with high confidence.

## Experiment 2

The goal of Experiment 2 was to test the hypothesis that congruent sounds, even when they are not relevant to the current task, improve memory by supporting the encoding of details from the episodic event. Results of Experiment 1 suggested that experiencing a visual object in the context of a semantically congruent sound produced better recognition memory than in the context of an incongruent sound, and exploratory analyses suggested that the memory enhancement was specific to the highest level of confidence. Within the dual-process model framework, such a result could indicate improvement in recollection memory, but not familiarity. This may also explain why significant differences were not detected between the control condition and the congruent or incongruent conditions, because a null effect on familiarity would mitigate an effect driven by recollection-based recognition when the outcome measure includes influences of both. In Experiment 2, we test this hypothesis in a modified recognition task in which the inclusion of additional confidence levels allowed us to obtain formal estimates of recollection and familiarity using the Dual-Process Signal Detection (DPSD) model (Yonelinas, 1994).

### Method

#### Participants

One hundred and thirteen students (99 identified as female and 32 identified as male, *M*_*age*_ = 20.11 years) from the University of California, Davis, participated in exchange for partial course credit. Participants were excluded under the same exclusion criteria that were pre-registered criteria for Experiment 1, namely, below chance accuracy on the encoding or recognition task and based on responses to the debriefing survey. We excluded five participants due to low accuracy, and 62 due to debriefing survey responses. The debriefing survey for this experiment included two additional questions regarding comprehension of the recognition task because it was more complex than the task used for Experiment 1. Participants were excluded if they did not fully understand the task or if they did not use the entire range of response options. We used the same target sample size as in Experiment 1, and data were collected until we reached 66 participants post-exclusion.

#### Materials

All stimuli were identical to those used in Experiment 1.

#### Procedure

As in Experiment 1, participants completed separate encoding and recognition blocks online via personal computers through the online stimulus presentation software Testable (https://www.testable.org/).

##### Encoding block

The encoding block in Experiment 2 was identical to Experiment 1; however, because there was no interaction between Visual Noise Condition and Auditory Condition in Experiment 1, all images in the encoding task for Experiment 2 were overlaid with visual noise instead of half as in Experiment 1. Visual stimuli were counterbalanced across the three, within-subjects Auditory Conditions.

##### Recognition block

Immediately following the encoding block, participants completed a visual-only surprise recognition task. This task was almost identical to the recognition task in Experiment 1, except for the response options. On each trial, a visual stimulus was presented for 400 ms, and participants could respond by clicking on buttons corresponding to “recollect,” “definitely old,” “probably old,” “unsure,” “probably new,” or “definitely new.” Participant instructions included a description and example of the difference between a “recollect” response and any “old” response, explaining that “recollect” should only be pressed if the participant was sure that they had seen the item before *and* they could recollect some qualitative information about the encoding event, such as their feelings about the item or what they thought about when they initially saw it.

##### Debriefing survey

After the encoding and recognition blocks, participants completed the debriefing survey, which was similar to Experiment 1, and was also used to exclude participants whose testing environment or stimulus presentations were not of adequate quality. To ensure that participants understood the recognition task, the debriefing survey included a question asking whether participants understood when they were supposed to press the “recollect” button, and a free-response question asking for an example of information they used to judge an item as recollected rather than definitely old (see OSM for full list of questions).

#### Data analysis

To compare overall memory performance between Auditory Conditions, we calculated AUC from observed ROCs. To directly assess effects of Auditory Conditions on recollection- and familiarity-based recognition, we fit equal variance signal detection models to the observed ROC data in line with the DPSD model to compare model parameters associated with these constructs (Yonelinas, 1994). We also analyzed responses to the open-ended debriefing survey prompt asking participants to report an example of information they used to base their “recollect” responses on. Mean accuracy (% correct) across conditions for the encoding and recognition tasks can be found in Table 2. It should be noted that “unsure” responses were treated as incorrect for calculating accuracy. Raw data files and analysis code for this experiment are publicly available on the Open Science Framework (https://osf.io/5uz24/).

**Table 2:** Mean accuracy for the encoding and recognition tasks, and mean Dual-Process Signal Detection (DPSD) model parameters for overall recognition memory (area under the curve; AUC), recollection (y-intercept), and familiarity (*d’*) for items in each condition for Experiment 2. Chance performance is 50% accuracy for both encoding and recognition tasks

Auditory condition	Task accuracy (% correct)		DPSD model parameters		
	Encoding task	Recognition task	AUC	y-intercept	*d’*
Congruent	0.81(0.39)	0.79(0.41)	0.85(0.09)	0.26(0.22)	1.54(0.73)
Control	0.80(0.40)	0.74(0.44)	0.82(0.10)	0.17(0.20)	1.44(0.70)
Incongruent	0.81(0.39)	0.74(0.44)	0.82(0.10)	0.18(0.18)	1.44(0.60)

Standard deviations are shown in parentheses

##### ROC analysis

**Fig. 3:**
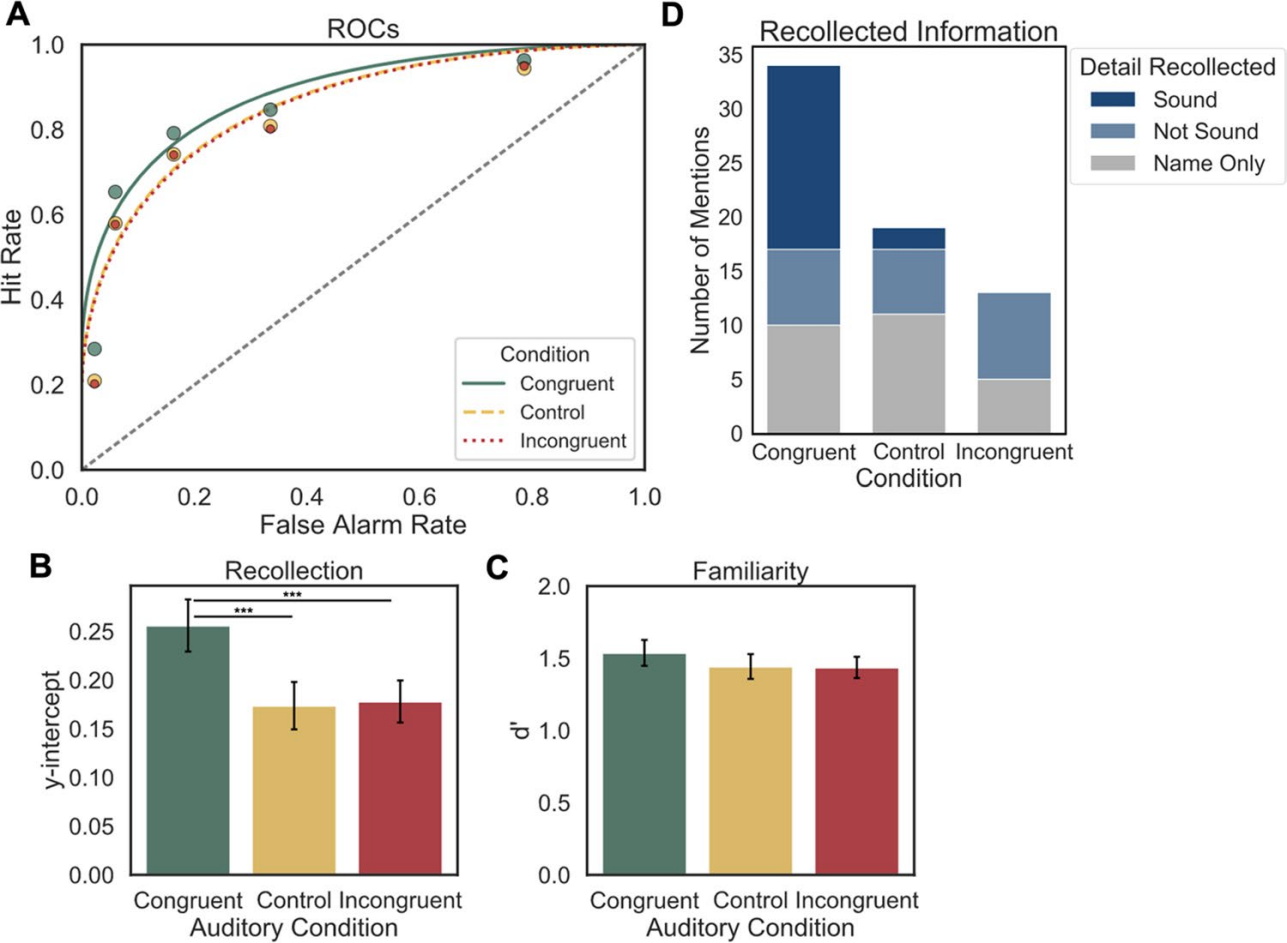
**a** The average observed receiver operating characteristic curves (ROCs; points) for each Auditory Condition from Experiment 2 and correspondin*g* Dual-Process Signal Detection (DPSD) equal-variance signal detection model functions. Overall memory performance (area under the curve; AUC) is greater for congruent than for incongruent or control items. **b** Average *y-intercept* for DPSD ROC curves between Auditory Conditions. Recollection is greater for congruent than incongruent or control items. **c** DPSD model-derived *d’* for each Auditory Condition. No significant differences between conditions. **d** Mentions of each type of recollected detail from the debriefing survey for items from each condition. Responses that did not mention a specific item (“Nonspecific” responses) are not included in the figure. All error bars denote standard error

Cumulative hit and false alarm rates were calculated for the observed ROCs just as in Experiment 1, though the response scale was larger for Experiment 2 (in line with previous DPSD studies), so the leftmost point corresponds to the hit and false alarm rates for trials on which participants responded “recollect,” the second point from the left reflects the hit and false alarm rates for trials on which participants chose either “recollect” or “definitely old,” and so on. For statistical analyses, individual ROCs were constructed for each participant at each level of Auditory Condition, and the points in Fig. 3a reflect the average observed hit and false alarm rates for these groups across participants. DPSD models were fit to each participant’s ROCs as they were in Experiment 1, and the average ROC model for each group is shown in Fig. 3a. To compare overall differences in memory performance between conditions, AUC was calculated for each participant’s observed ROCs in each Auditory Condition and compared via one-way ANOVA and Bonferroni-corrected post hoc pairwise t-tests. Parameter estimates derived from DPSD model-based ROCs were used to compare two constructs of interest from the dual-process model of recognition memory, namely the *y-intercept*, which estimates recollection, and *d’*, which estimates familiarity. In the DPSD model, the y-intercept estimates the hit rate when the false alarm rate is equal to 0, making it a threshold measure of memory that represents recollection. Model-derived *d’* measures hit rates relative to false alarm rates across the entirety of the curve, which quantifies the contribution of familiarity. These estimates were also compared via individual one-way ANOVAs and Bonferroni-adjusted post hoc pairwise t-tests. Bayes factors were also computed for pairwise comparisons and interpreted in the same manner as the previous experiment.

##### Debriefing questionnaire analysis

To perform an exploratory assessment of the details that were recollected about objects on trials for which participants respond with “recollect,” we coded the open-ended responses to the debriefing survey for mentions of specific items and/or features recollected from the encoding task (see OSF page for all responses and their categorizations). Responses that referred to objects and their accompanying sound were labeled as “Sound” recollections (e.g., *“I remembered the dog because it was shown along with a ‘bark’ sound”*). Responses that referred to the objects and other aspects of the encoding experience were labeled as “Not Sound” recollections (e.g., *“I remembered the elephant because elephants are my mom’s favorite animal”*). Responses that only listed the name of the visual object were labeled as “Name Only” recollections (e.g., *“bird”*). The responses to each object were summed across the Auditory Condition to which the object was encoded for each participant. In cases where a participant mentioned multiple items, all items were included in the analysis, so the total number of responses exceeds the number of items included in the analysis. For the analysis, we compared the count of items mentioned from each Auditory Condition (congruent, incongruent, control), and the detail given as part of the response (“Sound,” “Not Sound,” and “Name Only”). A small proportion of the responses included a detail that was recollected without mentioning a specific item (e.g., *“I pressed ‘recollect’ if I remembered the sound that played with an item in the first task”*). These responses are categorized as “Nonspecific” because they do not contain explicit object labels and are discussed separately from the responses that did mention specific items, and are not included in Fig. 3d.

### Results

#### ROC analysis

##### AUC

A one-way repeated-measures ANOVA revealed a significant effect of Auditory Condition on AUC, *F*(2, 130) = 9.84, *p* < 0.001, *η*_*p*_^*2*^ = 0.13, and post hoc t-tests showed that memory performance was better for congruent items than control or incongruent items, *t*(65) = 3.94, *p* < 0.001; *t*(65) = 3.86, *p* < 0.001, and Bayes factors suggest very strong evidence for both findings (*BF*_10_ = 112.20; *BF*_10_ = 86.51). (Table 2). AUC was not significantly different between control and incongruent items, *t*(65) = -0.41, *p* = 1.00, and the Bayes factor provides moderate evidence for the null hypothesis (*BF*_01_ = 6.67). These results show that recognition was better for items in the congruent condition than the control or incongruent conditions.

##### Recollection and familiarity

A one-way repeated-measures ANOVA showed a significant effect of Auditory Condition on recollection (*y-intercept*), *F*(2, 130) = 11.10, *p* < 0.001, *η*_*p*_^*2*^ = 0.15 (Fig. 3b), with higher y-intercepts for items in the congruent condition than in the control condition or incongruent condition, but no significant difference between items in the control and the incongruent conditions, *t*(65) = 4.03, *p* < 0.001; *t*(65) = 4.65, *p* < 0.001; *t*(65) = -0.20, *p* = 1.00, and Bayes factors provided very strong evidence for the findings in the first two comparisons (*BF*_10_ = 148.66; *BF*_10_ = 1084.05), and moderate evidence for the null hypothesis in the latter (*BF*_01_ = 7.14). A one-way repeated-measures ANOVA did not show a significant effect of Auditory Condition on familiarity (*d’*), *F*(2, 130) = 1.02, *p* = 0.37 , *η*_*p*_^*2*^ = 0.02 (Fig. 3c). These results confirm our hypothesis and converge with the exploratory analysis in Experiment 1, showing that improvements in memory for the congruent Auditory Condition were due to better recollection-based recognition memory. Interestingly, we found no effect of Auditory Condition on familiarity, suggesting that the effect of an auditory event was specific to encoding mechanisms that improve recollection.

##### Debriefing questionnaire

Out of a total of 76 responses, ten (13.2%) were “Nonspecific,” and of these, three mentioned that they chose “recollect” if they remembered the sound that an item was paired with but neither the object nor the sound was explicitly named; the other seven mentioned non-sound details, but also did not provide explicit labels for the objects. The 66 responses that did specifically name items included 34 named congruent items, 19 control items, and 13 incongruent items. Fifty percent of the named congruent items included “Sound” details, while only 10.5% of control items mentioned “Sound” details, and none of the incongruent items included “Sound” details (Fig. 3d). There were a similar number of responses in each of the three conditions mentioning “Not Sound” details (Fig. 3d).

## Experiment 3

Experiment 2 found that a semantically congruent multisensory event led to better recollection-based recognition memory, indicating that they produced a more detailed memory of the encoded event. Based on this finding, we would expect not only better recognition of the visual object, but also better memory for the association between the visual object and the sound. We tested this hypothesis in Experiment 3 by altering the memory test to ask participants in which Auditory Condition they experienced each visual object. Although the recall task was expected to be more difficult, fewer items were included in this experiment, and therefore two levels of Visual Noise were included to prevent possible ceiling or floor effects.

### Method

#### Participants

Seventy-six students (65 identified as female, ten identified as male, and one identified as other, *M*_*age*_ = 19.17 years) from the University of California, Davis, participated in exchange for partial course credit. Participants were excluded under our pre-registered exclusion criteria, namely, below chance accuracy on the encoding or recognition task and based on responses to the debriefing survey. Ten participants were excluded due to low accuracy, and zero due to debriefing survey responses. We used the same target sample size as in Experiments 1 and 2, and data were collected until we reached 66 participants post-exclusion.

#### Materials

All stimuli were identical to those used in Experiments 1 and 2. However, in the recognition task, instead of 90 new items, there were only 30 in order to keep the number of items equal across each of the four response options, for a total of 120 items in the recognition task (see OSM).

#### Procedure

As in Experiments 1 and 2, participants completed separate encoding and recognition blocks online via personal computers through the online stimulus presentation software Testable (https://www.testable.org/).

##### Encoding block

The encoding task in Experiment 3 was identical to the encoding task in Experiment 1. We included the visual noise manipulation from Experiment 1 as a precaution to ensure that ceiling or floor effects would be avoided (see Fig. 1a).

##### Recognition block

Immediately following the encoding block, participants completed a visual-only surprise recognition task. This task was similar to the tasks in Experiments 1 and 2, with a few exceptions. Ninety “old” items were mixed with 30 “new” items, and on each trial, participants were asked to indicate whether the object was originally presented with a sound that was the same as the object (congruent), different from the object (incongruent), a meaningless, white-noise sound (control), or if the object was new (see Fig. 1b).

##### Debriefing survey

After the encoding and recognition blocks, participants completed the debriefing survey, which was the same as the survey used for Experiment 1 and was also used to exclude participants whose testing environment or stimulus presentations were not of adequate quality (see OSM for full list of questions).

#### Data analyses

To assess memory for the auditory encoding condition in Experiment 3, we calculated the sensitivity index *d’* for hits and false alarms for old items in each Auditory Condition. In this experiment, a hit occurred when an old item was attributed to the correct encoding condition (congruent, control, or incongruent sound), and a false alarm occurred when an old item was attributed to the incorrect encoding condition. Our preregistered analysis plan included a repeated-measures ANOVA to compare raw memory accuracy (percent correct) between Auditory and Visual Noise Conditions, though we deviated from this plan because the false alarms in each condition were unevenly distributed across response options. Specifically, when participants saw an old item that had initially been presented in the control or incongruent conditions, they most often incorrectly attributed these items as belonging to the congruent condition during encoding. As such, this potentially inflated the raw accuracy of the congruent condition, so we used the measure of *d’* to avoid this potential confound. It should be noted that new items had false alarms that were evenly distributed across the congruent, control, and incongruent responses, suggesting that the response bias was unique to old items. We performed a 2 (Visual Noise: visual noise vs. no visual noise) × 3 (Auditory Condition: congruent, control, incongruent) repeated-measures ANOVA on the *d’* performance index, and Bonferroni-adjusted post hoc pairwise t-tests. Bayes factors were computed for pairwise comparisons. Additionally, mean accuracy (% correct) for the encoding and recognition tasks can be found in Table 3.

**Table 3:** Mean accuracy for the encoding and recognition tasks and mean *d’* for each Auditory Condition and Visual Noise Condition from Experiment 3. Chance performance is 50% accuracy for the encoding and 25% accuracy for the recognition task

Auditory condition	Encoding task (% Correct)		Recognition task (% Correct)		Recognition task *d’*	
	No visual noise	Visual noise	No visual noise	Visual noise	No visual noise	Visual noise
Congruent	0.89(0.31)	0.82(0.38)	0.65(0.48)	0.55(0.50)	0.97(0.66)	0.88(0.58)
Control	0.88(0.32)	0.79(0.41)	0.22(0.42)	0.19(0.40)	0.52(0.48)	0.56(0.42)
Incongruent	0.85(0.36)	0.78(0.41)	0.28(0.45)	0.24(0.43)	0.51(0.46)	0.47(0.40)

Standard deviations are shown in parentheses

### Results

**Fig. 4:**
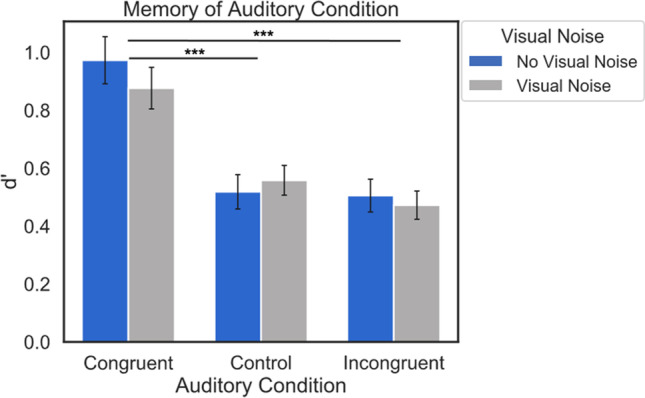
Average *d’* for each Visual Noise and Auditory Condition. Error bars denote standard error of the mean. Memory was better for the sound played during encoding for items in the congruent condition than the incongruent or control conditions

A repeated-measures ANOVA on memory for the encoding condition (*d’*) showed no significant interaction between Visual Noise and Auditory Condition, *F*(2, 130) = 0.98, *p* = 0.38, and no significant effect of Visual Noise, *F*(1, 65) = 0.41, *p* = 0.53, *η*_*p*_^*2*^ = 0.006. However, there was a significant effect of Auditory Condition, such that memory for the auditory encoding condition was better for items in the congruent encoding condition than the control or incongruent conditions, but no difference between control and incongruent conditions, *F*(2, 130) = 29.95, *p* < 0.001, *η*_*p*_^*2*^ = 0.32; *t*(65) = 6.18, *p* < 0.001; *t*(65) = 6.78, *p* < 0.001; *t*(65) = -0.85, *p* = 1.00, with Bayes factors provided very strong evidence for the finding from the first two comparisons (*BF*_10_ = 2.73 x 10^5, *BF*_10_ = 2.73 x 10^6), and moderate evidence for the null hypothesis for the last comparison (*BF*_01_ = 5.26) (Table 3, Fig. 4). These results suggest that in addition to better memory for the visual stimulus, the presence of a congruent sound facilitates the retrieval of the task-irrelevant auditory stimulus, even though participants were not aware that their memory would be tested for either.

### Discussion

The goal of the present research was to investigate whether congruent multisensory presentation facilitates visual recognition memory by supporting recollection or familiarity-based recognition. Our results replicated previous findings (Lehmann & Murray, 2005; Thelen et al., 2015), even with tests of incidental memory and when hit rates were compared across multiple false alarm rates. More importantly, consistent with our hypothesis, our results provide the first evidence that memory improvement for semantically congruent audiovisual pairs specifically promotes recollection. We also showed that learning object-congruent sounds not only improved memory for the task-relevant visual object, but also for the sounds themselves despite being task-irrelevant and the memory tests being completely unexpected. Together, our experiments demonstrate that the presence of an object-congruent sound at encoding increases the likelihood that an episodic memory for an object will be formed and later recollected.

Our findings also suggest that this memory benefit is due to the integration of semantically congruent information into the encoded object representation, and that improvement to perceptual fluency during encoding cannot alone explain our findings. In Experiment 1, visual noise impaired encoding and recognition performance overall, but the impact was equivalent for congruent, incongruent, and control audiovisual object pairs. If the benefit for audiovisual pairs in recognition memory stemmed from increased perceptual fluency, the effect of visual noise should have been smaller for congruent pairs, but that was not the case. However, we cannot rule out the possibility that the specific conditions used in our experiments may have reduced the effect of perceptual fluency. For example, Chen and Spence (2010) used much briefer image presentations (27 ms) than we did (400 ms), and found benefits of multisensory processing on perceptual fluency for object identification (see also Driver & Noesselt, 2008). Experiment 3 similarly showed overall poorer encoding and recognition accuracy with visual noise across conditions, and there was no main effect of Visual Noise when accounting for response biases using *d’*. Overall, the lack of interaction between Auditory Condition and Visual Noise in these studies suggests that multisensory processing did not facilitate recognition memory merely by increasing perceptual fluency.

Experiment 2 suggests that the multisensory memory effect is driven by a mechanism that facilitates the storage, and later recollection, of details from the encoded event, particularly for the sound itself. This extends previous research on semantic congruency of word-pairs on recollection – rather than familiarity – to more naturalistic stimuli (Bein et al., 2015). In both the Bein et al. (2015) study and ours, the recollection benefit may be specific to retrieval of the accessory information presented at encoding (the adjective in their study and the sound in the present study). Analysis of the debriefing survey in Experiment 2 supports this possibility, and future research will be needed to investigate whether a congruent sound at encoding leads to better recollection because it helped reinstate visual object details or the context, or because it reduced processing demands associated with encoding consistent information along for more visual details to be encoded in the first place. Evidence that redundant multisensory signals provide neural and behavioral benefits over redundant unisensory signals in cats (Alvarado et al., 2007; Gingras et al., 2009) and humans (Laurienti et al., 2004) suggests that crossmodal accessory information could provide support over and above accessory unisensory information, but such comparisons have yet to be made in memory studies. Regardless of whether there is more than one mechanism underlying the multisensory memory benefit, the present study presents an ecologically valid situation in which congruent semantic information facilitates later recollection-based memory.

Experiment 3 showed that the relation between the objects and sounds was more likely to be remembered when the sound was congruent, despite being task-irrelevant during encoding. This is consistent with research showing that attention spreads from a task-relevant to a task-irrelevant stimulus in another modality that corresponds semantically (Fiebelkorn et al., 2010; Molholm et al., 2007; Zimmer et al., 2010). For example, Molholm et al. (2007) showed serial audiovisual presentations of objects that were congruent or incongruent, and had participants perform an N-back task on stimuli in either the visual or the auditory modality. Processing of the stimulus in the ignored modality was enhanced when it was semantically congruent with the stimulus in the attended modality, as indexed by the SN ERP component. In the current studies, attention likely also spread from visual items to semantically congruent sounds, and this attentional enhancement of multiple pieces of object-related information may be responsible for the recollection-specific benefit (Craik, Govoni, Naveh-Benjamin, & Anderson, 1996; Greene et al., 2021; Troyer & Craik, 2000).^1^

This interpretation fits well within a predictive coding framework, which posits that the brain maintains an internal model of the environment that generates predictions about the environment and sensory inputs are either confirmatory or produce an error signal (Friston, 2010; Friston & Kiebel, 2009). Talsma (2015) proposed that congruent crossmodal stimuli produce a signal with low prediction error, resulting in a stronger memory trace and less effortful encoding than an incongruent crossmodal stimulus that would produce an error signal and require a model update. Such a mechanism could explain our results because attention to two congruent constituents of a multisensory stimulus would be expected and reinforce the same internal representation, leading the object to be more readily bound into an episodic memory than if attention is divided when the audiovisual event is incongruent.^2^

In summary, the present studies expand upon research on the memory benefits of congruent multisensory events by showing that a visual object encoded with a congruent sound is more likely to be recognized later based on detailed recollection. While our evidence indicates that memory for the sound supports recollection-based recognition of the visual object, future studies will be necessary to determine whether memory for other details, such as specific visual features or its context, are also improved. Nevertheless, the present study illustrates how multisensory events can produce a qualitative shift in the encoding of episodic events.

## Supplementary information

ESM 1 (DOCX 565 kb)

## Footnotes

1 It is possible that this enhanced attention to object-specific sensory information could specifically facilitate memory for that visual object and sound itself, thereby incurring a cost to memory for other aspects of the encoded event, though future studies will be needed to understand whether the present effect comes at any sort of impairment.

2 Across our experiments, we did not find memory impairments for items presented with incongruent sounds relative to control sounds as previous studies have, which may have been due to the relatively high proportion of incongruent trials included in our experiments (see Thelen et al., 2015).
