# Supplementary material for: Object memory is multisensory: Task-irrelevant sounds improve recollection
Source: Psychon Bull Rev. 2022 Sep 27;30(2):652–65. doi: 10.3758/s13423-022-02182-1 (PMC10040470; doi:10.3758/s13423-022-02182-1)

Supplemental Information for:

**Title: Object memory is multisensory: Task-irrelevant sounds improve recollection**

**Authors:** Shea E. Duarte^1,2^, Simona Ghetti^1,2^, & Joy J. Geng^1,2^

1. Center for Mind and Brain, University of California Davis, Davis, CA, 95618

2. Department of Psychology, University of California Davis, Davis, CA, 95618

**Table 1.** Visual object stimuli for Experiments 1-3.

| **Experiment** | **Old/New** | **Size** | **Object** | **Category** |
| --- | --- | --- | --- | --- |
| 1, 2, 3 | Old | Small | hammer | tool |
|  |  |  | saw | tool |
|  |  |  | bird | animal |
|  |  |  | hen | animal |
|  |  |  | frog | animal |
|  |  |  | rat | animal |
|  |  |  | cat | animal |
|  |  |  | bat | animal |
|  |  |  | smartphone | common |
|  |  |  | keyboard | common |
|  |  |  | bell | instrument |
|  |  |  | snake | animal |
|  |  |  | stapler | household |
|  |  |  | scissors | household |
|  |  |  | camera | common |
|  |  |  | drill | tool |
|  |  |  | basketball | recreation |
|  |  |  | blender | household |
|  |  |  | book | household |
|  |  |  | bowling pin | recreation |
|  |  |  | chick | animal |
|  |  |  | clock | household |
|  |  |  | cup | household |
|  |  |  | straw | household |
|  |  |  | flute | instrument |
|  |  |  | coins | common |
|  |  |  | laptop | common |
|  |  |  | hairdryer | household |
|  |  |  | harmonica | instrument |
|  |  |  | kettle | household |
|  |  |  | key | household |
|  |  |  | lighter | tool |
|  |  |  | light switch | household |
|  |  |  | maracas | instrument |
|  |  |  | matches | tool |
|  |  |  | pencil | household |
|  |  |  | Ping-Pong paddle | recreation |
|  |  |  | tape | household |
|  |  |  | soda can | household |
|  |  |  | spray bottle | household |
|  |  |  | teapot | household |
|  |  |  | tennis racket | recreation |
|  |  |  | toaster | household |
|  |  |  | wine bottle | household |
|  |  |  | xylophone | instrument |
| 1, 2, 3 | Old | Large | elephant | animal |
|  |  |  | dog | animal |
|  |  |  | horse | animal |
|  |  |  | pig | animal |
|  |  |  | cow | animal |
|  |  |  | bear | animal |
|  |  |  | tiger | animal |
|  |  |  | motorcycle | vehicle |
|  |  |  | guitar | instrument |
|  |  |  | jet | vehicle |
|  |  |  | car | vehicle |
|  |  |  | piano | instrument |
|  |  |  | drum | instrument |
|  |  |  | cymbal ride | instrument |
|  |  |  | goat | animal |
|  |  |  | deer | animal |
|  |  |  | leopard | animal |
|  |  |  | wolf | animal |
|  |  |  | arcade game | recreation |
|  |  |  | axe | tool |
|  |  |  | baseball bat | recreation |
|  |  |  | billiards | recreation |
|  |  |  | bicycle | vehicle |
|  |  |  | train | vehicle |
|  |  |  | door | common |
|  |  |  | washer | household |
|  |  |  | crocodile | animal |
|  |  |  | goose | animal |
|  |  |  | penguin | animal |
|  |  |  | boat | vehicle |
|  |  |  | microwave | household |
|  |  |  | printer | common |
|  |  |  | sink | household |
|  |  |  | skateboard | vehicle |
|  |  |  | sled | recreation |
|  |  |  | sword | common |
|  |  |  | toilet | household |
|  |  |  | toilet brush | household |
|  |  |  | Bow/arrow | recreation |
|  |  |  | chair | household |
|  |  |  | anvil | tool |
|  |  |  | filing cabinet | household |
|  |  |  | fireplace | household |
|  |  |  | golfclub | recreation |
|  |  |  | helicopter | vehicle |
| 1,2,3 | New | Mixed | wrench | tool |
|  |  |  | whale | animal |
|  |  |  | watering can | household |
|  |  |  | walkie | common |
|  |  |  | scooter | vehicle |
|  |  |  | shark | animal |
|  |  |  | salamander | animal |
|  |  |  | record player | instrument |
|  |  |  | rhino | animal |
|  |  |  | rabbit | animal |
|  |  |  | fridge | household |
|  |  |  | fish | animal |
|  |  |  | crab | animal |
|  |  |  | frypan | household |
|  |  |  | camel | animal |
|  |  |  | butterfly | animal |
|  |  |  | bus | vehicle |
|  |  |  | radio | common |
|  |  |  | air hockey | recreation |
|  |  |  | briefcase | common |
|  |  |  | calculator | common |
|  |  |  | candle | household |
|  |  |  | football | recreation |
|  |  |  | hoe | tool |
|  |  |  | lamp | household |
|  |  |  | microphone | instrument |
|  |  |  | notepad | common |
|  |  |  | plant | household |
|  |  |  | pliers | tool |
|  |  |  | scorpion | animal |
| 1, 2 | New | Mixed | pen | common |
|  |  |  | screwdriver | tool |
|  |  |  | snail | animal |
|  |  |  | soccer ball | recreation |
|  |  |  | spider | animal |
|  |  |  | spoon | common |
|  |  |  | table | household |
|  |  |  | turtle | animal |
|  |  |  | umbrella | common |
|  |  |  | water bottle | common |
|  |  |  | whisk | household |
|  |  |  | eraser | common |
|  |  |  | octopus | animal |
|  |  |  | screw | tool |
|  |  |  | toucan | animal |
|  |  |  | zebra | animal |
|  |  |  | bed | household |
|  |  |  | cake stand | household |
|  |  |  | closet | household |
|  |  |  | couch | household |
|  |  |  | espresso maker | household |
|  |  |  | tree | common |
|  |  |  | stove | household |
|  |  |  | barrel | common |
|  |  |  | bucket | common |
|  |  |  | crowbar | tool |
|  |  |  | rope | tool |
|  |  |  | scale | tool |
|  |  |  | tv | common |
|  |  |  | bedside table | household |
|  |  |  | mirror | common |
|  |  |  | mocha pot | household |
|  |  |  | bathtub | household |
|  |  |  | spatula | household |
|  |  |  | basket | common |
|  |  |  | box | common |
|  |  |  | duffel | common |
|  |  |  | giftbox | common |
|  |  |  | pallet | tool |
|  |  |  | shelf | common |
|  |  |  | suitcase | common |
|  |  |  | trash can | common |
|  |  |  | treasure chest | common |
|  |  |  | extinguisher | tool |
|  |  |  | tire | common |
|  |  |  | chess | recreation |
|  |  |  | cone | common |
|  |  |  | streetlight | common |
|  |  |  | hydrant | tool |
|  |  |  | ladder | tool |
|  |  |  | mailbox | common |
|  |  |  | street sign | common |
|  |  |  | beach chair | recreation |
|  |  |  | fork | household |
|  |  |  | life ring | tool |
|  |  |  | outlet | common |
|  |  |  | shovel | tool |
|  |  |  | surfboard | recreation |
|  |  |  | clipboard | common |
|  |  |  | globe | common |

**Table 2.** Incongruent sounds used across all 3 experiments, and the objects they were paired with in each counterbalanced version of the experiment.

| **Sound** | **Incongruent Visual Pairing 1** | **Incongruent Visual Pairing 2** | **Incongruent Visual Pairing 3** |
| --- | --- | --- | --- |
| ambulance | drill | key | hammer |
| bagpipe | basketball | lighter | saw |
| bee | blender | light switch | bird |
| bongo | book | maracas | hen |
| broom | bowling pin | matches | frog |
| chainsaw | chick | pencil | rat |
| chimp | clock | ping pong paddle | cat |
| comb | cup | microwave | elephant |
| cricket | deer | printer | dog |
| donkey | leopard | sink | horse |
| fencing | wolf | skateboard | pig |
| fire alarm | arcade game | sled | cow |
| foghorn | axe | sword | bear |
| glass | baseball bat | toilet | tiger |
| gong | billiards | toilet brush | motorcycle |
| grate | cup straw | tape | bat |
| guinea pig | flute | soda can | smartphone |
| gun | coins | spray bottle | keyboard |
| harp | laptop | teapot | bell |
| ice cube | hairdryer | tennis racket | snake |
| morse | harmonica | toaster | stapler |
| music box | kettle | wine bottle | scissors |
| razor | bicycle | xylophone | camera |
| roulette | train | bow arrow | guitar |
| snapper | door | chair | jet |
| sealion | washer | anvil | car |
| trampoline | crocodile | filing cabinet | piano |
| trumpet | goose | fireplace | drum |
| whip | penguin | golfclub | cymbal ride |
| whistle | boat | helicopter | goat |

**Table 3.** The number of old and new exemplars per category and the recognition accuracy (% correct) for Experiments 1 and 2. Note that “unsure” responses in Experiment 2 were considered incorrect.

| Category | Old Items | New Items | Difference | Recognition Accuracy Exp 1 | Recognition Accuracy Exp 2 |
| --- | --- | --- | --- | --- | --- |
| **common misc.** | 8 | 31 | -23 | 0.81(39) | 0.69(0.46) |
| **tool** | 7 | 14 | -7 | 0.77(42) | 0.63(0.45) |
| **recreation** | 10 | 6 | 4 | 0.87(34) | 0.82(0.38) |
| **animal** | 22 | 16 | 6 | 0.81(0.40) | 0.77(0.42) |
| **vehicle** | 8 | 2 | 6 | 0.76(43) | 0.72(0.45) |
| **household** | 26 | 19 | 7 | 0.76(42) | 0.67(0.47) |
| **instrument** | 9 | 2 | 7 | 0.81(39) | 0.82(0.38) |

*Standard deviations are shown in parentheses

**Table 4.** Debriefing questions for experiments 1-3

| Experiment | Question | Response Options |
| --- | --- | --- |
| 1, 2, 3 | Was the volume on your computer enabled throughout the entire first task? | Yes; No |
|  | Did you adjust your volume at any time during the experiment? | Yes; No |
|  | How would you describe the volume of the sounds during the first task? | Quiet; Loud; Just right; I did not hear sounds |
|  | Did you use external speakers, in-ear headphones, over-ear headphones? | External speakers; In-ear headphones; Over-ear headphones; Other; I did not hear sounds |
|  | How would you describe the environment in which you completed the study? | Quiet; Mostly quiet; Somewhat noisy; Very noisy |
|  | Did you experience distractions during the study? | Yes, major distractions; Yes, minor distractions; No |
|  | How much effort did you put into the experiment? | Not any; Not very much; Some effort; A lot of effort |
|  | How difficult did you find task 1? | Not difficult; A little difficult; Very difficult |
|  | How difficult did you find task 2? | Not difficult; A little difficult; Very difficult |
|  | In the first task, did you ever experience a lag or gap between when the picture was shown and when the sound started? | Yes, a couple of times; Yes, often or always; No; I did not hear sounds |
| 2 | In the second task, did you understand when you were supposed to press the “Recollect” button? | Yes, definitely; Yes, I think so; Not sure; Not at all |
|  | Please give an example of something you recalled about an object on a trial where you pressed the “Recollect” button. | Free-response |

**Table 5.** Example visual stimuli. In the encoding tasks, images with and without visual noise were used for Experiments 1 and 3, and only images with visual noise were used for Experiment 2. Post-stimulus masks were presented immediately after images in the encoding task across experiments. In the memory test of all three experiments, the images were never overlaid with visual noise.


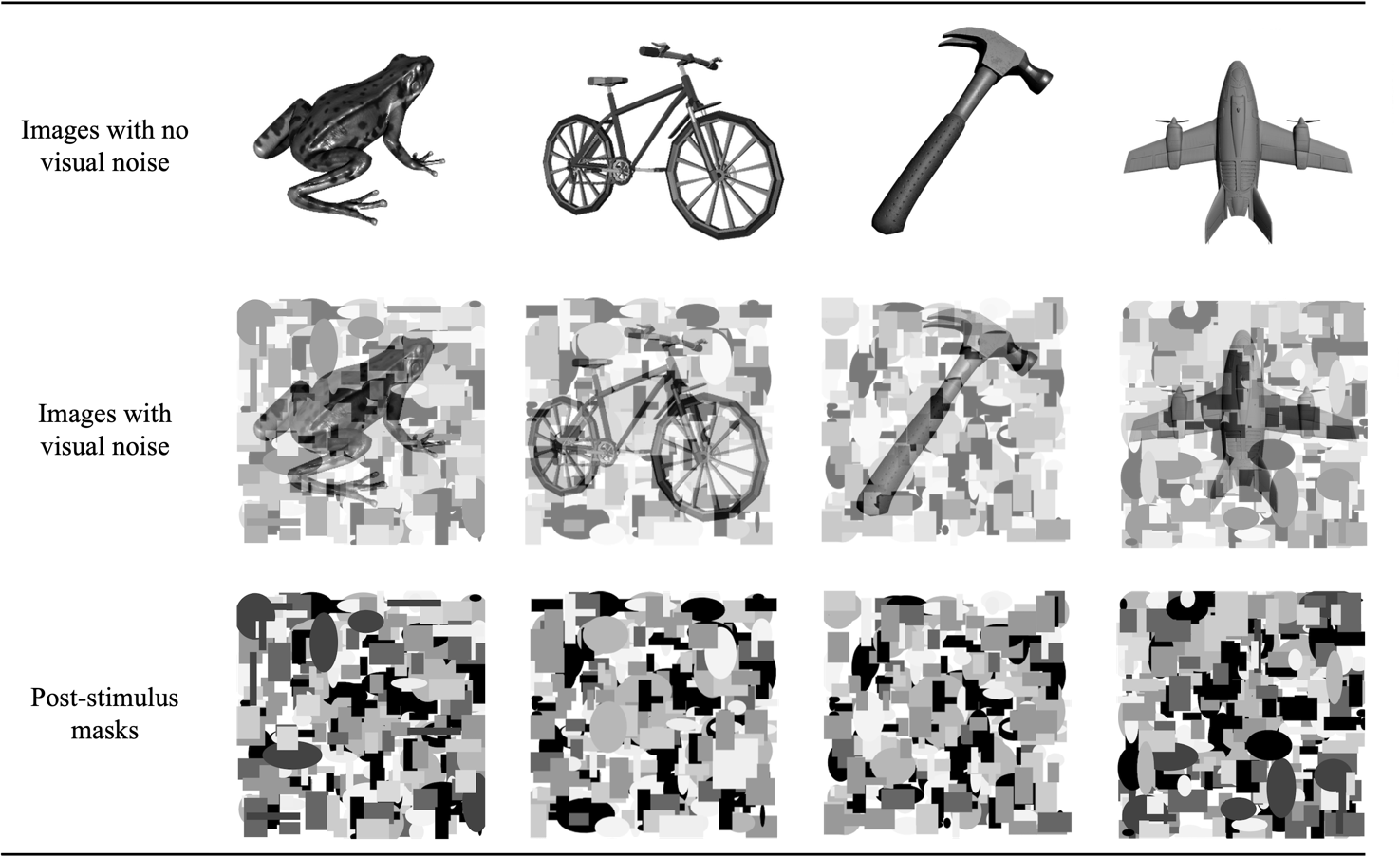

Supplement: Supplementary file 1 — (DOCX 565 kb) [file 13423_2022_2182_MOESM1_ESM.docx]
